# Molecular dating of caprines using ancient DNA sequences of *Myotragus balearicus*, an extinct endemic Balearic mammal

**DOI:** 10.1186/1471-2148-5-70

**Published:** 2005-12-06

**Authors:** Carles Lalueza-Fox, Jose Castresana, Lourdes Sampietro, Tomàs Marquès-Bonet, Josep Antoni Alcover, Jaume Bertranpetit

**Affiliations:** 1 Unitat de Biologia Evolutiva, Facultat de Ciències de la Salut i de la Vida, Universitat Pompeu Fabra, Doctor Aiguader 80, 08003 Barcelona, Spain; 2 Unitat d'Antropologia, Dept. Biologia Animal, Facultat de Biologia, Universitat de Barcelona, Avda. Diagonal 645, 08028 Barcelona, Spain; 3 Department of Physiology and Molecular Biodiversity, Institut de Biologia Molecular de Barcelona, CSIC, 08034 Barcelona, Spain; 4 Institut Mediterrani d'Estudis Avançats de les Illes Balears (CSIC-UIB), Cta. de Valldemosa km 7.5, 07071 Ciutat de Mallorca, Spain

## Abstract

**Background:**

*Myotragus balearicus *was an endemic bovid from the Balearic Islands (Western Mediterranean) that became extinct around 6,000-4,000 years ago. The *Myotragus *evolutionary lineage became isolated in the islands most probably at the end of the Messinian crisis, when the desiccation of the Mediterranean ended, in a geological date established at 5.35 Mya. Thus, the sequences of *Myotragus *could be very valuable for calibrating the mammalian mitochondrial DNA clock and, in particular, the tree of the Caprinae subfamily, to which *Myotragus *belongs.

**Results:**

We have retrieved the complete mitochondrial cytochrome *b *gene (1,143 base pairs), plus fragments of the mitochondrial 12S gene and the nuclear 28S rDNA multi-copy gene from a well preserved *Myotragus *subfossil bone. The best resolved phylogenetic trees, obtained with the cytochrome *b *gene, placed *Myotragus *in a position basal to the *Ovis *group. Using the calibration provided by the isolation of Balearic Islands, we calculated that the initial radiation of caprines can be dated at 6.2 ± 0.4 Mya. In addition, alpine and southern chamois, considered until recently the same species, split around 1.6 ± 0.3 Mya, indicating that the two chamois species have been separated much longer than previously thought.

**Conclusion:**

Since there are almost no extant endemic mammals in Mediterranean islands, the sequence of the extinct Balearic endemic *Myotragus *has been crucial for allowing us to use the Messinian crisis calibration point for dating the caprines phylogenetic tree.

## Background

*Myotragus balearicus *[1] was an extremely modified caprine, endemic of the Balearic Islands, characterized by a series of unusual apomorphies developed throughout more than five million years of insular evolution [2-7]. *Myotragus *disappeared from Mallorca between 3,700 and 2,040 years BC [8], probably after the arrival of the first humans to the Islands. The morphological peculiarities of *Myotragus*, including extreme size reduction, a single ever growing lower incisor, modified limb bones and frontal eyes [2], makes it difficult to clarify its taxonomic position [9].

In two previous studies [10,11], we obtained bits of the mtDNA cytochrome *b *gene from two different *Myotragus *bone specimens from Mallorca island, one found in cova Estreta (Pollença) and the other found in Cova des Gorgs (Escorca). Although the quick radiation of all the Caprinae [12] and the short cytochrome *b *fragment retrieved (338 bp) made the previous analysis difficult, our data indicated that *Myotragus *was genetically close to the sheep (*Ovis*) group.

There is a general agreement that the continental ancestor of the Balearic bovid possibly settled in Mallorca and Menorca during the Messinian period. The Balearic Islands were last connected to the continent during the Messinian regression, when the Mediterranean basin was dried out, allowing faunal exchanges between the islands and the continental lands [13,14]. The opening of the Strait of Gibraltar 5.35 Mya definitely isolated the Balearic Islands and promoted the beginning of the independent evolution of *Myotragus *[2]. The *Myotragus *lineage spreads from the Pliocene to the Holocene, and includes in Mallorca five chronospecies: *M. pepgonellae, M. antiquus*, *M. kopperi*, *M. batei *and *M. balearicus*. No absolute datings are available for the oldest species, *M. pepgonellae*, although a chronology of Lower Pliocene has been proposed; a paleomagnetic data of about 2.6 My is available for *M. antiquus *remains [2]. Apart from two shrews from the islands of Crete and Sicily, respectively, no other Pliocene endemic mammal persists in Mediterranean islands [15,16]. Thus, the accurately dated vicariant event that isolated *Myotragus *from their continental relatives constitutes a unique opportunity to use the sequences of *Myotragus *for calibrating the DNA molecular clock of caprines. Previously, only dates outside caprines (the emergence of the family Bovidae 18.5 Mya), or dates that are too recent for accurate estimations (common ancestry of some domestic sheep breeds a few thousand years ago) could be used [17,18]. In addition, since the molecular clock is not perfect, it is necessary to use as much sequence information as possible to obtain reliable date estimates. For this purpose, we decided to retrieve the complete cytochrome *b *gene and some other genetic markers of *Myotragus*; to do this, we used a newly excavated *Myotragus *bone, obtained in 2002 from Cova des Gorgs, that looked macroscopically very well preserved and therefore was suggestive of DNA survival. We designed different sets of overlapping primers for retrieving the complete cytochrome *b *gene, as well as a 305 bp fragment from another mtDNA gene, the 12S. In addition, we retrieved a short fragment of a nuclear gene, 28S rDNA, from the same extract. The only precedent of nuclear DNA retrieval from warm climates is, to our knowledge, the analysis of a ground sloth coprolite from the south of United States [19].

Our results confirm that *Ovis *is the sister group of *Myotragus*. We also show that the initial radiation of caprines occurred 6.2 ± 0.4 Mya, more recent than the date obtained in other analyses that used very different calibration points [17,18]. Finally, we show that the cytochrome *b *sequences of alpine and southern chamois, until recently considered to be subspecies, have been separated 1.6 ± 0.3 Mya, much longer than previously suspected.

## Results and discussion

### Ancient DNA sequences

The complete 1,143 nucleotides of the cytochrome *b *gene were retrieved in seven overlapping fragments (see primers in Table 1), including the 338 base pairs section between positions 14,312 and 14,649 (using *Ovis *as reference sequence) already retrieved in a previous work [11] and a section of 85 bp replicated in Oxford (between positions 14,399 and 14,483) (Table 2). Two discrepancies with the previous consensus sequences were observed, a T in position 14,635 and a T in 14,638. The first one was already reported as heteroplasmic in the cloned PCR product in [11], where it was present in five up to ten clones sequenced. The latter was not found in our previous study; therefore, it can correspond to a silent polymorphism within the *Myotragus *population or to a DNA damage involving a rare A to T change in that particular PCR reaction. All cytochrome *b *fragments were routinely cloned (not shown), although very few heteroplasmies were detected in the direct sequencing, attributable to both the exceptional preservation of the sample and the short lengths of the fragments; consequently, the error rate in the clones (number of nucleotide differences/1,000 base pairs) was very low (< 2 per thousand base pairs). Considering the overlapping of the primers and the fragment replicated, 404 bp (around 35% of the cytochrome *b *gene) have been determined from more than one PCR. It cannot be discarded that DNA damage could have affected some positions, although it is unlikely that this would significantly alter the phylogenetic inferences.

**Table 1:** Primer sequences used in this study for different mtDNA and nuclear genes; L and H refers to light and heavy strand, respectively, and numbers refers to the 3' position of the *Ovis *mtDNA sequence.

**Primer sequence**	**Amplicon length**
Cytochrome *b *gene	
L14136 5'-GCTTGATATGAAAAACCATCGTTG-3' H14313 5'-TGTGTCGGATGTATAGTGTATTG-3'	176 bp
L14310 5'-ATCCTAACAGGCCTATTCCT-3' H14481 5'-CCGATGTTTCATGTTTCTAGGA-3'	170 bp
L14475 5'-CGAGGCCTGTACTACGGATC-3' H14650 5'-AACTGAGAATCCGCCTCAG-3'	174 bp
L14631 5'-GCTATCCCATACATTGGAAC-3' H14813 5'-GTATARTARGGGTGAAATGG-3'	181 bp
L14792 5'-TCCAACAACCCCTCAGGAATTC-3' H14987 5'-TTGATCGTARGATTGCGTATGC-3'	194 bp
L14973 5'-CCTCACATCAAACCCGAATG-3' H15159 5'-TCCTCCAATTCATGTGAGTG-3'	185 bp
L15152 5'TTCTGAATCCTAGTAGCCGACC-3' H15327 5'-TGCAGTCATCTCCGGTTTACAAGAC-3'	174 bp
12 S gene	
L599 5'-CTCAAAGGACTTGGCGGTGC-3' H673 5'-GAAGATGGCGGTATATAGAC-3'	73 bp
L671 5'-TCACCAATCCTTGCTAATAC-3' H805 5'-AATGGCTTTCGTATTAAATT-3'	133 bp
L733 5'-AACAAGAGTAAGCTCAATCA-3' H891 5'-CGGTGTGTGCGTGCTTCATG-3'	157 bp
28 S gene	
L28S 5'-GGTCGTCCGACCTGGGTATA-3' H28S 5'-TCTAATCATTCGCTTTACCGGAT-3'	96 bp

**Table 2:** *Myotragus *sequence of the complete mtDNA cytochrome *b *(between positions 14,159 and 15,301), aligned with the *Ovis *sequence. Dots indicate sequence identity.

OVIS MYOTRAGUS	14159	ATG ATC AAC ATC CGA AAA ACC CAC CCA CTA ATA AAA ATT GTA AAC AAC GCA TTC ATT GAT ... .C. ... T.. ... ... ... A.. ... ... ... ... ... ... ... ... ... ... ... ..C
OVIS MYOTRAGUS	14219	CTC CCA GCT CCA TCA AAT ATT TCA TCA TGA TGA AAC TTT GGC TCT CTC CTA GGC ATT TGC ... ... ..C ... ... ..C ..C ... ... ... ... ... ..C ... ..C ... ... ... G.C ...
OVIS MYOTRAGUS	14279	TTA ATT TTA CAG ATT CTA ACA GGC CTA TTC CTA GCA ATA CAC TAT ACA CCC GAC ACA ACA ... ..C ... ..A ..C ... ... ... ... ... ... ... ... ... ... ... T.. ... ... ...
OVIS MYOTRAGUS	14339	ACA GCA TTC TCC TCT GTA ACC CAC ATT TGC CGA GAC GTG AAC TAT GGC TGA ATT ATC CGA ... ... .G. ... ... ..C G.. ..T ... ... ... ... ..A ... ... ... ... ... ... ...
OVIS MYOTRAGUS	14399	TAT ATA CAC GCA AAC GGG GCA TCA ATA TTT TTT ATC TGC CTA TTT ATG CAT GTA GGA CGA ... ... ..T ... ... ..A ... ..C ... ... ..C G.. ... ... ... ... ..C ..G ... ..G
OVIS MYOTRAGUS	144590	GGC CTA TAT TAT GGA TCA TAT ACC TTC CTA GAA ACA TGA AAC ATC GGA GTA ATC CTC CTA ... ... ..C ..C ... ... ..C ..T ... ... ... ... ... ... ... ... A.. ... ... ...
OVIS MYOTRAGUS	14519	TTT GCG ACA ATA GCC ACA GCA TTC ATA GGC TAT GTC TTA CCA TGA GGA CAA ATA TCA TTC ..C A.A ... ... ..T ... ... ... ... ..T ..C ..T ... ... ... ... ... ... ..T ..T
OVIS MYOTRAGUS	14579	TGA GGA GCA ACA GTT ATT ACC AAC CTC CTT TCA GCA ATT CCA TAT ATT GGC ACA AAC CTA ... ... ... ..C ... ..C ... ... ... ..C ... ..T ..C ... ..C ... ..A ..C ..T ..T
OVIS MYOTRAGUS	14639	GTC GAA TGA ATC TGG GGA GGA TTC TCA GTA GAC AAA GCT ACC CTC ACC CGA TTT TTC GCC ..A ... ... ... ..A ... ... ... ... ... ... ..G ..C ... ... ..A ... ..C ... ..T
OVIS MYOTRAGUS	14699	TTT CAC TTT ATT TTC CCA TTC ATC ATC GCA GCC CTC GCC ATA GTT CAC CTA CTC TTC CTC ... ... ..C ..C C.. ... ..T ..T ... ... ... ... ... ... ..C ... ... ..A ... ...
OVIS MYOTRAGUS	14759	CAC GAA ACA GGA TCC AAC AAC CCC ACA GGA ATT CCA TCG GAC ACA GAT AAA ATT CCC TTC ... ... ... ... ... ... ... ... T.. ... ... ... ..A ... G.. ..C ... ..C ..A ..T
OVIS MYOTRAGUS	14819	CAC CCT TAT TAC ACC ATT AAA GAC ATC CTA GGT GCT ATC CTA CTA ATC CTC ATC CTC ATG ... ..C ... ... ... ... ... ... ... ... ..C ATG ..A ... ... ..T T.A ... ... ...
OVIS MYOTRAGUS	14879	CTA CTA GTA CTA TTC ACG CCT GAC TTA CTC GGA GAC CCA GAC AAC TAC ACC CCA GCA AAC ... ... ... ... ... ..A ..A ... C.. ... ... ... ... ..T ..T ..T ..A ... ..C ...
OVIS MYOTRAGUS	14939	CCA CTT AAC ACT CCC CCT CAC ATC AAA CCT GAA TGA TAC TTC CTA TTT GCG TAC GCA ATC ... ..C ... ..A ... ... ... ... ... ..C ... ... ... ... ... ..C ..A ... ... ..T
OVIS MYOTRAGUS	14999	TTA CGA TCA ATC CCT AAT AAA CTA GGA GGA GTC CTC GCC CTA ATC CTC TCA ATC CTA GTC C.. ... ... ..T ..C ..A ... ... ... ... ..T ..A ... ... G.. ... ... ..T ..G A..
OVIS MYOTRAGUS	15059	CTA GTA ATT ATA CCC CTC CTC CAT ACA TCA AAG CAA CGG AGC ATA ATA TTC CGA CCA ATC ... ..G C.. ... ..T ..A ... ..C .A. ..C ..A ... T.A ... ... ... ... .AG ... ..T
OVIS MYOTRAGUS	15119	AGT CAA TGC ATA TTC TGA ATC CTA GTA GCC GAC CTA TTA ACA CTC ACA TGA ATT GGA GGA ... ... ..T C.. G.. ... ..T T.. ... ..A ... ... C.. ... ... ... ... ... ... ..C
OVIS MYOTRAGUS	15179	CAG CCA GTT GAA CAC CCC TAC ATC ATT ATT GGA CAA CTA GCA TCT ATT ATA TAT TTC CTT ... ... ... ... ... ... ..T ..T ... ... ... ..G ... ... ... ... ... ... ... ...
OVIS MYOTRAGUS	15239	ATC ATT CTA GTC ATA ATA CCA GTA GCT AGC ATC ATC GAA AAC AAC CTC CTA AAA TGA AGA ... ... ... ... ... ... ... ..G ..G ... .C. ... ... ... ... ... ... ... ... ...
OVIS MYOTRAGUS	15299	CAA ...

The putative presence of nuclear mtDNA insertions is very unlikely, since we proceed designing the L primers for the next fragment in the sequence already retrieved from the previous fragment, and the H primers in a consensus Caprinae sequence. The 12S gene sequence (305 base pairs) was retrieved in three overlapping fragments (see primers in Table 1). The sequences found in 12S showed several differences from those of extant Caprinae, a fact that points to its authenticity.

The PCR of the 28S nuclear gene yielded a very faint band around 140 base pairs and was subsequently cloned and sequenced. In addition, DNA from tissue samples from domestic goat (*Capra aegagrus*), southern chamois (*Rupicapra pyrenaica*) and domestic sheep (*Ovis aries*) was extracted in a separated laboratory, and the same 28S fragment was determined. The 96 base pairs sequence obtained from *Myotragus *is similar to that found in other Caprinae (Table 3), and clearly different to the human one and to the cow, a suggested source of contamination because of the use of BSA (bovine serum albumine) in ancient DNA amplifications. Moreover, no other Bovids, extinct or living, had been analyzed in the same laboratory when the extraction, amplification, cloning and sequencing of the *Myotragus *genes was undertaken; therefore, the 28S sequence seems to be endogenous of *Myotragus*. The cloning of the 28S PCR product (Table 3) showed that two of the sequences (about 20% of the clones) were human contaminants; this accounts for the noise observed in the direct sequencing of the PCR product. Most likely, the human DNA comes from handling of the *Myotragus *bones during its excavation and posterior morphological study. Nevertheless, the error rate in the clones is relatively low (3.07 per thousand base pairs), a figure within the range found in some modern specimens and well preserved ancient remains, like the moas [20].

**Table 3:** *Myotragus *clones and consensus sequence of the nuclear gene 28S rDNA, aligned with sequences of *Capra*, *Ovis *and *Rupicapra *obtained in this study (including *Bos *as a reference sequence).

BOS	GGGGCGAAAGACTAATCGAACCATCTAGTAGCTGGTTCCCTCCGAAGTTTCCCTCAGGATAGCTGGCGCTCTCGCA-AACGC-ACAG--ACCC-ACGCAGTTTT
M clone1	............................................................................---...----.-A....-..........
M clone2	............................................................................---...----.-A....-..........
M Clone3	..........................................................................T.---...----.-A....-..........
M Clone4	............................................................................---...----.-A....-..........
M Clone5	............................................................................---...----.-A....-..........
M Clone6	.......................................T..................................T.---...----.-A....-..........
M Clone7	............................................................................---...----.-A....-..........
M Clone8	................................................C...........................---...----.-A....-..........
M CONSENSE	............................................................................---...----.-A....-..........
CAPRA	............................................................................-...C--------....-..........
OVIS	............................................................................-------------....-..........
RUPICAPRA	............................................................................G.-..-G...AG-....-..........

Variation in length is characteristic of 28S gene; size variations are due to expansions or contractions of variable segments in 10–12 positions within the gene, where variation does not interfere with ribosomal function [21]. The sequences were aligned by eye; due to the high interspecific variation in 28S, no definite alignment is possible and therefore, the phylogenetic information obtained from this gene is limited. However, the retrieval of a nuclear gene indicates the quality of the DNA of the sample used. Furthermore, it opens new possibilities of research for assessing the phylogenetic relationships of *Myotragus *and for retrieving nuclear genes with phenotypic implications.

### Phylogeny of the Caprinae subfamily and the position of *Myotragus *in the tree

**Figure 1:**
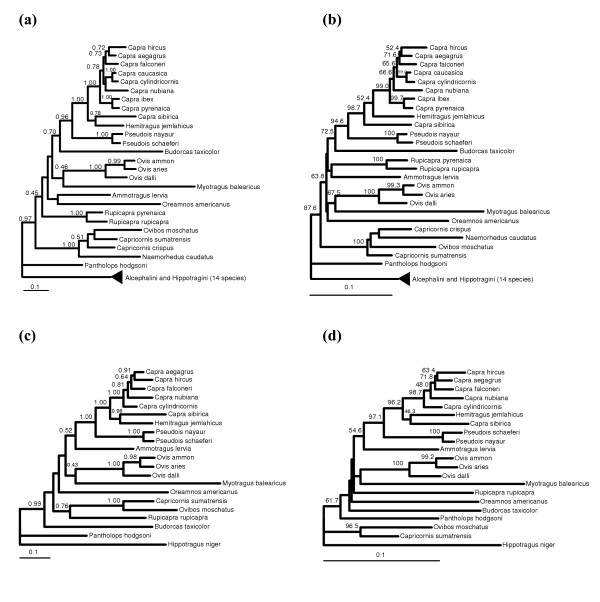
Bayesian tree (a) and maximum-likelihood tree (b) of the entire cytochrome *b *of all Caprinae species in databases. Bayesian tree (c) and maximum-likelihood tree (d) of the concatenated cytochrome *b *plus a 12S fragment of all Caprinae species in databases with both sequences. Bootstrap per cent values higher than 40% (maximum-likelihood tree) and support values bigger than 0.4 (Bayesian tree) are depicted in the nodes. The scale bar represents 0.1 substitutions/site.

We included *Pantholops hodgsoni *in the phylogenetic analysis since previous analysis as well as ours indicate that this species is clearly associated to other members of the subfamily Caprinae. As an outgroup, we used 14 members of the tribes Alcephalini and Hippotragini (see Methods) previously detected as the closest members within the Bovidae [17,22]. The cytochrome *b *and cytochrome *b *+ 12S Bayesian and maximum-likelihood trees (Figure 1) showed a topology roughly similar to that found in previous studies on Caprinae phylogeny (e.g., [22]). Recently, it has been reported that the *Budorcas *cytochrome *b *sequence available at GenBank was in fact a chimaera that included a fragment of *Ovis *sequence, and a new sequence for this species was provided [17]. Crucially, in previous works *Myotragus *formed a quite stable clade with *Budorcas *and *Ovis *that is not maintained anymore, with *Budorcas *now clustering in a different position.

Examination of the cytochrome *b *trees (Figure 1a and 1b) shows that some of the clades previously found are present in these trees, specially *Myotragus*+*Ovis *(67.5% in the maximum-likelihood trees), *Capricornis*+*Ovibos*+*Naemorhedus *(100% in the maximum-likelihood tree) and *Hemitragus*+*Pseudois*+*Capra *(94.6% in the maximum-likelihood tree). The position of *Hemitragus*, that precludes the monophyly of the genus *Capra*, is problematic, as some authors have already noticed [18]. Some species show unstable positions in the trees, particularly the genera *Budorcas*, *Ammotragus*, *Pantholops*, *Rupicapra *and *Oreamnos*. The problematic position of these taxa has been already reported on extant species studies (see, for instance [22,23]). However, our tree, although still with low bootstrap values, has been able to better resolve the phylogenetic position of *Myotragus*, which is basal to the *Ovis *clade; the support value for the *Myotragus*+*Ovis *grouping is now 0.46 in the Bayesian tree and the bootstrap value 67.5% in the maximum-likelihood tree, respectively [11].

The trees with the cytochrome *b *+ 12S fragment (305 base pairs) did not significantly improve the phylogeny (Figure 1c and 1d); the *Capricornis*+*Ovibos *and *Hemitragus*+*Pseudois*+*Capra *clades are well supported by bootstrap analysis, while the support of the cluster of *Myotragus*+*Ovis *is reduced with respect to the cytochrome *b *sequence alone. This discrepancy can be attributed to the small number of informative positions added by the 12S fragment together with the reduced number of species that could be used due to the unavailability of the 12S sequence in many species.

### Evolutionary rates and diversification of Caprinae

The complete resolution of the Caprinae phylogeny cannot be achieved with the cytochrome *b *gene alone, as some species of the subfamily (specially of the genera *Budorcas*, *Oreamnos*, *Rupicapra*, *Ammotragus *and *Pantholops*) still have an unstable position. It is likely that the lack of resolution in the basal branches is due to the existence of a very quick initial radiation in the Caprinae subfamily. To test this, we performed a likelihood ratio test for zero branch-length of all branches of the tree, that showed that there are nine branches with p > 0.05 (and therefore, not significantly different from 0). Five of them belong to the basal branches of the Caprinae tree, thus supporting the hypothesis of a very fast initial radiation of this group. Additionally, the plot of grow of lineages of an ultrametric tree (see below) also supports this fast initial radiation.

Being *Myotragus *a small caprine, its long branch in the maximum-likelihood tree previously detected with a cytochrome *b *fragment [11] was attributed to an earlier age of first reproduction and a shorter generation time in *Myotragus *than in other wild caprines [11]. This long branch is also present in the trees of the complete cytochrome *b *(Figure 1a and 1b). However, recent estimates indicate that *Myotragus *was actually bigger than previously believed, with a weight ranging between 15 and 25 kg for the smallest adult specimens and between 40 and 60 kg for the larger specimens [24]. Therefore the reason for this long branch cannot be due to its presumed extreme reduced size (and the consequences that this involves). On the other hand, the hypothesis that tries to explain differences in evolutionary rates as due to differences in metabolic rate or generation time (both correlated to body mass) was not supported in the analysis of cytochrome *b *evolutionary rates of a wide set of mammalian species [25]. It is thus more likely that this relatively long branch of *Myotragus *in the Caprinae tree is mainly a stochastic phenomenon.

### Dating of the Caprinae phylogeny

**Figure 2:**
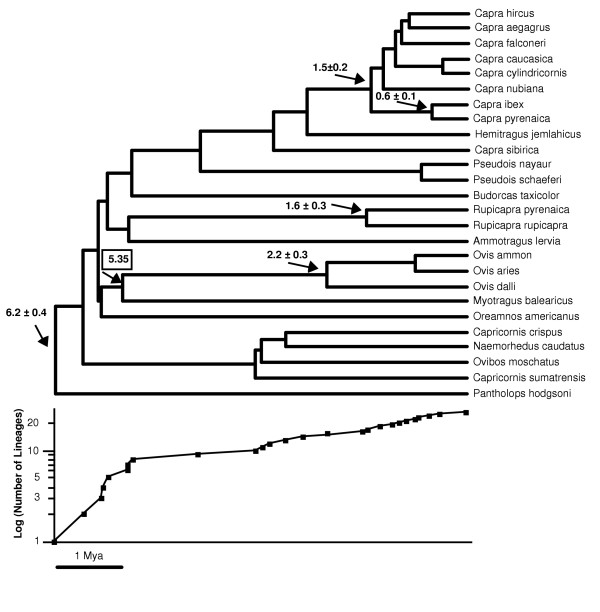
Ultrametric tree of Caprinae derived from the cytochrome *b *maximum-likelihood tree. The geological date of the isolation of the *Myotragus *lineage in the Balearic islands 5.35 Mya, that was used as a calibration point, is highlighted. Calculated dates and standard deviations are given for some important splits in the tree. A lineages through time plot is shown under the tree. The number of lineages in each node is show in logarithmic scale. The horizontal scale bar represents 1 Mya for both the tree and the lineages through time plot.

Due to the endemicity of *Myotragus *[2], it can be hypothesized that this lineage diverged from the continental species 5.35 million years ago (Mya), when the desiccation of the Mediterranean ended [13,14] and the *Myotragus *ancestors became isolated in the Balearic Islands. In principle, this would be a minimum age for the node in the tree, but we consider likely that the isolation of the Balearic Islands was a vicariant event responsible for the split of the *Myotragus *and continental lineages, so that we can use this date as a fixed age in the Caprinae cytochrome *b *tree (Figure 2). The dating results for the maximum-likelihood and Bayesian trees are very similar and only the former are shown. We have calculated ultrametric trees using the Langley-Fitch method [26] as well as the penalyzed likelihood method with several smoothing factors [27] implemented in the r8s program. The cross-validation procedure to explore the fidelity with which these different methods explain the branch length variation in the tree indicates that the Langley-Fitch method is slightly better for our data set and therefore we used this method in the calculations described next (although dates obtained were very similar using penalyzed likelihood with a log_10 _of the smoothing factor of 2; not shown). In addition, we have performed a parametric bootstrap procedure, in which we simulated alignments from the maximum-likelihood tree and re-calculated dates from the same maximum-likelihood topology with branch lengths optimized for each simulated alignment. This allowed us to evaluate the stochastic errors of date estimates associated to sampling a finite number of alignment positions [28]. In a different parametric bootstrap procedure, we reconstructed new trees from each simulated alignment and used them for time estimation (instead of only the original topology) and therefore errors associated with finite number of positions as well as with tree reconstruction imperfections are estimated [28]. The standard deviations and 95% confidence intervals for most date estimates were very similar for both parametric bootstrap procedures (Table 4), indicating that most of the errors in the present data set are associated to the finite sample sequence rather than to tree errors. In the following, standard deviations given refer to the parametric bootstrap using only the original topology for calculating dates.

**Table 4:** Dates in Mya, standard deviations, and 95% intervals (in parenthesis) obtained with parametric bootstrap using the original topology (Single topology) or new tree calculations (Several topologies) for each simulation of the mtDNA cytochrome *b *of Caprinae. The calibration is based on the isolation of *Myotragus *in the Balearic Islands at 5.35 million years ago.

Lineage	Single topology	Several topologies
*Capra*	1.5 ± 0.2 (1.2 – 1.9)	1.5 ± 0.2 (1.1 – 1.9)
*Capra ibex*/*C. pyrenaica*	0.6 ± 0.1 (0.4 – 0.9)	0.6 ± 0.1 (0.3 – 0.8)
*Rupicapra pyrenaica*/*R. rupicapra*	1.6 ± 0.3 (1.1 – 2.1)	1.5 ± 0.3 (1.1 – 2.1)
*Ovis*	2.2 ± 0.3 (1.7 – 2.8)	2.1 ± 0.3 (1.6 – 2.8)
Root	6.2 ± 0.4 (5.5 – 7)	6.1 ± 0.5 (5.4 – 7.2)

The ultrametric trees calculated by the Langley-Fitch method applied to the cytochrome *b *maximum-likelihood tree showed that the separation of the most divergent lineages within Caprinae (*Pantholops *and the rest of species) occurred 6.2 ± 0.4 Mya. These dates are more recent than a proposed Caprinae radiation 11 Mya based on different molecular calibrations (see Introduction) [17,18]. There is no general consensus from fossil data on the date of the start of the Caprinae radiation, that ranges from around 14 Mya [12] to a more recent Late Miocene origin [29], which would be more consistent with our results.

After the first split, the separation of the most basal lineages took place around or before 5.35 Mya (Figure 2), and therefore we can estimate that the main radiation of caprines occurred in less than one million years of evolution. A lineages through time plot, that reflects the number of lineages in each split point of the tree (bottom of Figure 2), indicates a fast growing of lineages during the first million years of evolution in comparison with the flatter slope in the rest of the tree. Although a few species are missing in this tree, all genera are covered and it is likely that the tree with all species will reveal the same clear steep slope at its base. This truly supports the existence of a quite fast initial radiation that led to the differentiation of nine clades (including the *Myotragus *lineage) that are at the base of all extant genera. In addition, this explains the difficulties we face in reconstructing the phylogenetic tree of this group.

Interestingly, the separation of the two chamois species (*Rupicapra*) and the beginning of the radiation of the main clade of goats (separation of the *Capra ibex *and *Capra pyrenaica *clade from the rest of species) occurred at 1.6 ± 0.3 Mya and 1.5 ± 0.2, respectively, according to the calibrated cytochrome *b *tree. This would imply that the beginning of the Pleistocene glaciations promoted the diversification of the main lineages of Caprinae species living in the Alps and other Eurasian mountainous regions. The deep split observed between the alpine and southern chamois, at 1.6 ± 0.3 Mya, is quite remarkable, since these two species have been considered subspecies until recently [30], and other molecular studies have given much more recent dates for the separation of these two species using restriction fragment length polymorphism of mitochondrial DNA [31] or microsatellites [32]. Their split is even older than that between *Capra pyrenaica *and *Capra ibex*, a pair of species with a similar geographic disjunction than the two chamois and traditionally recognized as different species, that separated 0.6 ± 0.1 Mya according to our data. To test whether the deep split observed between the two chamois was caused by particularly divergent cytochrome *b *haplotypes of chamois used in this study, we performed the calibrations with all sequences of chamois in databases, including shorter ones (five for *R. pyrenaica *and six for *R. rupicapra*), obtaining very similar dates (not shown). To analyze if the date of this split depended on the particularly long branch of *Myotragus*, we manually reduced this branch to the half, but we obtained again similar dates for all splits in the trees (1.7 Mya for the split between chamois). This is due to the smoothing of rates performed by the calibration methods used, that correct for the higher evolutionary rate of the *Myotragus *lineage. To account for the possibility that *Myotragus *could be more basal in the tree, we manually positioned *Myotragus *just after the separation of *Pantholops*. Then the split date of the two chamois was reduced, but only up to 1.4 Mya. This is in agreement with the parametric bootstrap analyses (Table 4), that indicate that the tree topology is not crucial in this calculation. This is surely due to the small internal branches involved in all different topologies arranging the basal species of the tree. Other works where several topologies were used for calculating dates have also shown that age estimates were largely insensitive to different phylogenies [28].

Finally, if *Myotragus *and *Ovis *had diverged in fact more recently and the ancestors of *Myotragus *had reached the Balearic Islands via a transmarine colonization after these islands became disconnected from the continent, as proposed for other vertebrates [32], all the dates in the tree could be more recent; however, this hypothesis is extremely unlikely in *Myotragus *from the paleontological evidence [24]. On the other hand, the use of evolutionary rates calculated from other sources, for example, the separation of humans and chimpanzees 5 – 7 Mya [34,35] combined with the genetic distance between their cytochrome *b *sequence, would lead to a separation of *Myotragus *and *Ovis *clades at 7.2 Mya. Although the use of an external calibration could be problematic, this reference would not support a transmarine colonization of *Myotragus *ancestors. Thus, the calibrated cytochrome *b *trees indicate that alpine and southern chamois have been separated much longer than previously thought, which would help explain the anti-hybridization mechanisms currently operating [30].

## Conclusion

In conclusion, this study is among the first ones in which mitochondrial as well as nuclear DNA has been retrieved from an extinct species from a warm location, in this case the Mediterranean area. Also, our research demonstrates the importance of ancient DNA in phylogenetics, since we have been able to use the isolation of *Myotragus *in the Baleric Islands as a vicariant event for dating the Caprinae phylogenetic tree. This is quite a unique opportunity for mammals because, contrary to other zoological groups such as insects, reptiles or amphibians, almost no extant endemic mammal remains in these Islands.

## Methods

A left tibiae (MNIB 60176) bone from *Myotragus balearicus *from Cova des Gorgs (Escorca, Mallorca), dated to 6,010-5,830 cal BC 2σ (Beta-177239) was chosen for DNA analysis because of its excellent external preservation. The remainder bone not used for analysis is held in the vertebrate collection in the Museu de la Naturalesa de les Illes Balears (MNIB, Palma de Mallorca); the present sample is approximately between 500 and 1,850 years younger than the other one from the same site previously analyzed [11].

DNA was extracted following procedures described elsewhere [11]. The sample was demineralized overnight with 0.5 M EDTA, incubated overnight with proteinase K and SDS, extracted with phenol-chloroform and concentrated and desalinized with centricon columns. Finally, the extract was purified with silica. The sample was extracted with blank extractions to monitor against contamination. Several fragments of mtDNA cytochrome *b *gene were amplified and sequenced until completing the whole gene (Table 1).

The cytochrome *b *sequences matched the partial sequence previously retrieved from another *Myotragus *bone from the same site [10]; multiple controls, such as independent replication of the results in two different laboratories and cloning of different overlapping PCR products, contributed to the authentication of the sequences. All the work was carried out in a dedicated ancient DNA laboratory, with UV lights, air-positive pressure and regular cleaning of surfaces with bleach. No signs of contamination were observed along the study in the extraction and PCR blanks; the sample was analyzed along with an Upper Paleolithic human remain [36] that yielded a human sequence. In addition, no other Bovid had ever been extracted in the same laboratory. One fragment of the cytochrome *b *was previously replicated in the Ancient Biomolecules Centre (Oxford), yielding the same sequence as in the present study. For the 12S gene, some primers were designed (Table 1) to match previous Caprinae sequences published [37].

Due to the exceptional quality of this extract, a pilot project was launched for trying to retrieve a fragment of a nuclear gene. The 28S rDNA was chosen because it is present in some hundreds of copies in the genome. The primers used to amplify the 28S rDNA were those designed [38] to be vertebrate-specific (Table 1).

Five microliters of extract were added to 20 μl PCR reactions, containing 1× reaction buffer, 1 unit of taq DNA polymerase, 2.5 mM of MgCl2, 25 pmol of each primer. Forty cycles of 1 min at 94°C, 1 min at 50°C and 1 min at 72°C were performed. PCR products were resolved in 1% low-melting agarose gels in a TA buffer; bands were excised from the gel and subjected to a second 30 cycles of PCR with limiting reagents. PCR products were cloned with the SureClone Ligation Kit (Pharmacia, Upsala, Sweden); inserts were sequenced with 3100 Gene Analyzer (Applied Biosystems).

Sequences have been deposited at the GenBank under accession numbers: AY380560, AY380561, AY380562, AY380563, AY894418, AY894419 and AY894420.

Several phylogenetic analyses were performed with the *Myotragus *sequences. We used its cytochrome *b *together with the whole cytochrome *b *of all Caprinae species available in databases (*Capra hircus*, *Capra aegagrus*, *Capra falconeri*, *Capra caucasica*, *Capra cylindricornis*, *Capra nubiana*, *Capra ibex*, *Capra sibirica*, *Hemitragus jemlahicus*, *Pseudois nayaur*, *Pseudois schaeferi*, *Ovis ammon*, *Ovis aries*, *Ovis dalli*, *Budorcas taxicolor*, *Myotragus balearicus*, *Rupicapra pyrenaica*, *Rupicapra rupicapra*, *Capricornis crispus*, *Ovibos moschatus*, *Naemorhedus caudatus*, *Pantholops hodgsoni*, *Oreamnos americanus*), plus the 987 base pairs sequence of *Capra pyrenaica *and the complete sequence of 14 members of Alcephalini and Hippotragini used as outgroups (*Addax nasomaculatus, Alcelaphus buselaphus, Alcelaphus lichtensteini, Beatragus hunteri, Connochaetes gnou, Connochaetes taurinus, Damaliscus lunatus, Damaliscus pygargus, Hippotragus equinus, Hippotragus niger, Oryx dammah, Oryx gazella, Oryx leucoryx *and *Sigmoceros lichtensteinii*); this high number of outgroups eliminates the possible biases of choosing a single one at random and partitions the longest branches, thus reducing the problem of long branch attraction [39]. The analyses considered a general time reversible (GTR) model with a proportion of invariable sites and a discrete gamma model with 6 categories to account for among site rate variation. This model was selected upon checking with Modeltest [40] that the set of all positions as well as the 1st, 2nd and 3rd codon positions favored the GTR model or a model close to it (GTR being the closest model in the phylogeny programs used here) and gamma plus invariable rates. Phylogenies were performed using Bayesian analysis implemented in the program MrBayes v. 3.0. [41]. For the calculations, independent models of sequence evolution were used for each codon position. Four chains of 4,000,000 trees were generated, sampling every 100th tree, with burning completed by the 20,000th tree; thus, 20,000 trees were used to estimate topologies and posterior probabilities of parameters. It was checked that the likelihood and all parameters were stationary after this burn-in. Maximum-likelihood analyses were performed with the program Phyml, version 2.4.4 [42]. The gamma parameter, the proportion of invariable sites and the nucleotide frequencies were estimated from the data. Two maximum-likelihood tree searches were made, one starting from a neighbor joining tree and another from the previously generated Bayesian tree, selecting the tree with the best likelihood (almost always the one starting from the Bayesian tree). To test the robustness of the tree clades, 1,000 bootstrap samples were performed.

Maximum-likelihood and Bayesian trees were similarly generated in a combination of the cytochrome *b *sequence with a 12S fragment, using the only 20 caprine species in which both 12S and cytochrome *b *were available at GenBank, and an outgroup. For the Bayesian tree, an independent GTR model was considered for the 12S partition. No tree was generated for the 28S fragment, due to irresolvable difficulties with the alignment.

To test the possibility of an explosive diversification of the Caprinae subfamily, we performed the likelihood ratio test for zero-length branches [43] from the cytochrome *b *data using PAUP 4,0 [44] and the same maximum-likelihood model as above.

To estimate absolute rates of molecular evolution with a relaxed molecular clock on the cytochrome *b *phylogenetic tree (and divergence times using the geological date of the isolation of the *Myotragus *lineage), the r8s v1.7 program was used [45]. Outgroup sequences were eliminated from the tree previous to the analysis of caprines. Smoothing of rate variation along the tree was performed with the Langley-Fitch [26] and penalized likelihood [27] methods. Sixteen smoothing factors with log_10 _from -2 to 5.5 were used for the penalized likelihood method. The lowest χ^2 ^cross-validation score, as calculated by r8s, was used to select the best method. To perform parametric bootstraps, 1,000 Monte Carlo simulations of alignments of the same length than the complete cytochrome *b *were generated with SeqGen [46] using the phylogenetic trees and model parameters previously obtained. A lineages through time plot of an ultrametric tree obtained with these methods was calculated with the program GENIE [47].

## Authors' contributions

CL-F and LS did the experimental work, JC carried out the phylogenetic and dating analyses, TM-B carried out some of the analyses, JAA provided the sample and critical feedback to CL-F, JB provided laboratory facilities. CL-F, JC and JB contributed to the writing of the manuscript.
